# The study of constructing evaluation indicators for the implementation of employee assistance programs in public sectors

**DOI:** 10.3389/fpsyg.2024.1339291

**Published:** 2024-04-24

**Authors:** Yin-Che Chen, Su-Ching Chung, Hui-Chuang Chu

**Affiliations:** ^1^Department of Educational Psychology and Counseling, National Tsing Hua University, Hsinchu City, Taiwan; ^2^Department of Personnel, Hsinchu County Government, Zhubei, Taiwan

**Keywords:** public sector, employee assistance programs, modified Delphi method, fuzzy analytic hierarchy process, ministry of labor

## Abstract

**Introduction:**

Employee assistance programs require resources and manpower of various natures across different types of public sector organization.

**Methods:**

This study began by outlining elements for comparing employee assistance programs’ evaluation criteria in four types of public sector organization on the basis of 22 service measures for such programs implemented by the Ministry of Labor in relation to three major aspects: work, life, and health. Elements of the evaluation criteria for public sector employee assistance programs were determined by surveying a panel of experts using the modified Delphi method. Last, the weight associated with the elements of evaluation criteria were calculated using the fuzzy analytic hierarchy process, and the criteria of four types of public sector organization were explored.

**Results:**

Data analysis indicated that the weight and priorities associated with elements of evaluation criteria for EAPs implemented by four types of public sector organization were not fully identical.

**Discussion:**

The results of this study suggest that, in terms of EAPs, the Directorate-General of Personnel Administration of the Executive Yuan should be pursuant to appropriate employee assistance programs provided by various public sector organizations according to the needs of their employees as well as the diverse objective conditions in which these organizations operate.

## 1 Introduction

Employee Assistance Programs (EAPs) are important for organizations as they provide support and services to employees, promoting their well-being and enhancing their performance. EAPs offer a range of services such as counseling, workshops, and support groups to help employees overcome personal and work-related obstacles that may affect their productivity. The implementation of EAPs should be tailored to the specific stage of organizational development, with work-related measures being crucial in all stages. EAPs can contribute to promoting mental wellness at the workplace, addressing personal concerns, and establishing a supportive environment. In addition, providing fair EAPs can enhance employee commitment and productivity, leading to the achievement of organizational goal (Azmi et al., 2022; Chellam and Divya, 2022; Chen et al., 2023).

The personnel administration authority of the public sector in Taiwan, the Directorate-General of Personnel Administration, Executive Yuan (2017), first implemented an EAP in 2003 and is currently evaluating EAPs in accordance with the “Effectiveness Evaluation Plan for EAPs Implemented by Competent Authorities of the Executive Yuan and Local Governments.” However, despite the implementation of the aforementioned plan, the Directorate-General has failed to closely integrate the plan with the EAP service measures, and the implementation results remain to be clearly outlined, thereby presenting a gap in practice. In addition, implementing EAPs requires investments of resource and labor, which differ among public sector organizations. However, the current evaluation plan for the evaluation indices and their weights in public sector EAPs have not been customized according to the diverse types and sizes of public sector organizations, resulting in improper follow-up evaluations for awards and failure to motivate public sector employees to promote EAPs. As a result, when public sector organizations promote and implement EAPs, implementors face confusion and increased administrative work and stress; this is the second gap in actual current practice (see Table 1).

**Table 1:** Weights of the overall measures in EAPs implemented by various organizations in the public sector.

Organization type	Executive yuan		Municipal government		County and city government		Others	
Criteria (weight/priority)	Work aspect	(0.26479/3)	Work aspect	(0.53225/1)	Work aspect	(0.51138/1)	Work aspect	(0.41049/1)
	Employee guidance	(0.19209/1)	Employee guidance	(0.26767/1)	Employee guidance	(0.22887/1)	Employee guidance	(0.1319/4)
	Job adaptation	(0.182/3)	Job adaptation	(0.21988/2)	Job adaptation	(0.12315/4)	Job adaptation	(0.1204/5)
	Job transition	(0.17253/4)	Job design	(0.10699/4)	Job design	(0.10208/7)	Job design	(0.10414/6)
	Career development	(0.18893/2)	Job transition	(0.13862/3)	Job transition	(0.12574/3)	Job transition	(0.04953/8)
	Retirement planning	(0.14411/5)	Career development	(0.09831/5)	Career development	(0.10901/6)	Career development	(0.14634/3)
	Crisis management	(0.12034,6)	Retirement planning	(0.08947/6)	Retirement planning	(0.1197/5)	Retirement planning	(0.16621/2)
			Resignation and career change	(0.07906/7)	Resignation and career change	(0.05397/8)	Resignation and career change	(0.06612/7)
					Crisis management	(0.13748/2)	Crisis management	(0.21537/1)
Criteria (weight/priority)	Daily life aspect	(0.31599/2)	Daily life aspect	(0.22071/3)	Daily life aspect	(0.28158/2)	Daily life aspect	(0.2/3)
	Financial and legal advice	(0.16849/3)	Financial and legal advice	(0.24682/1)	Financial and legal advice	(0.1571/3)	Financial and legal advice	(0.22713/1)
	Leisure and entertainment	(0.12739/6)	Leisure and entertainment	(0.12688/4)	Childcare and eldercare	(0.38034/1)	Leisure and entertainment	(0.10729/6)
	Family and marriage	(0.17361/2)	Family and marriage	(0.20917/2)	Life management	(0.12899/4)	Family and marriage	(0.16752/4)
	Childcare and eldercare	(0.16844/4)	Childcare and eldercare	(0.20406/3)	Interpersonal relationships	(0.22327/2)	Interpersonal relationships	(0.18226/2)
	Interpersonal relationships	(0.21378/1)	Life management	(0.10707/5)	Living assistance	(0.1103/5)	Insurance planning	(0.13505/5)
	Living assistance	(0.14828/5)	Interpersonal relationships	(0.10599/6)			Living assistance	(0.18075/3)
Criteria (weight/priority)	Health aspect	(0.41921/1)	Health aspect	(0.24704/2)	Health aspect	(0.20704/3)	Health aspect	(0.38956/2)
	Drug and alcohol abstinence	(0.12126/5)	Anxiety	(0.26707/1)	Drug and alcohol abstinence	(0.10933/5)	Anxiety	(0.17299/5)
	Anxiety	(0.27646/1)	Healthy diet	(0.1481/5)	Anxiety	(0.25721/2)	Healthy diet	(0.17528/4)
	Healthy diet	(0.18712/3)	Exercise and health maintenance	(0.15362/4)	Healthy diet	(0.14265/4)	Exercise and health maintenance	(0.19155/3)
	Exercise and health maintenance	(0.24082/2)	Stress management	(0.25443/2)	Exercise and health maintenance	(0.15898/3)	Stress management	(0.21304/2)
	Mental health	(0.17433/4)	Mental health	(0.17679/3)	Stress management	(0.33183/1)	Mental health	(0.24713/1)

Most EAP research has explored the private sector as the research subject and focused on the implementation and organizational performance or employee commitment and happiness, and relevant research on the priority ranking of the various elements of EAP service measures implemented by the Directorate-General remains scarce. However, effectiveness evaluation plans are closely related to the public sector EAPs and are a key medium and means that affect EAP implementation in the public sector. Therefore, this study used the service measures of an EAP implemented by the Ministry of Labor to determine the connotation of the EAP service measures of the public sector in order to construct evaluation indices for public sector EAPs and to bridge gaps in the literature.

### 1.1 Empirical research on employee assistance programs

Employee Assistance Programs (EAPs) are important tools for organizations in supporting their employees’ well-being and maximizing their potential (Al-Fayez and Goodman, 2024). EAPs provide various services and partnerships to help employees overcome personal and organizational obstacles that affect their productivity (Doran, 2022). The implementation and effectiveness of EAPs can vary across different types of organizations. For example, in U.S. federal agencies, employee assistance programs have a significant positive impact on organizational performance, especially when there is leadership support. In deposit money banks in Rivers State, Nigeria, employee assistance programs are found to enhance employee commitment, including affective, normative, and continuance commitment (Chen et al., 2023). In Taiwan, the implementation of EAPs in different stages of organizational development varies, with work-related measures being crucial in all stages and a shift toward health-related measures in later stages (Chen et al., 2021). In public sector organizations in Taiwan, the evaluation criteria and priorities for EAPs differ based on the organization type, with varying emphasis on aspects such as health, work, and crisis management.

Past research has demonstrated various aspects of employee assistance programs (EAPs), indicating that EAPs utilize appropriate intervention methods to identify, assess, and diagnose important issues that may affect employee job performance, actively addressing employees’ problems. Csiernik (2003) conducted research indicating that factors such as supervisor and coworker support, job characteristics, advocacy activities, as well as the confidentiality, professionalism, accessibility, and familiarity of EAP counseling, may influence employees’ utilization of EAPs. In recent years, numerous studies have investigated the relationship between EAPs and organizational factors, workload, turnover rates, and employee issues. The majority of research findings suggest that EAPs bring positive benefits to businesses or organizations. Below, I will elaborate on the organizational aspect, employee aspect, and public sector implementation, respectively.

#### 1.1.1 Organization

Lee et al. (2008) stated that organizations provide benefits such as assistance programs, health benefits, health care services, stress relief courses, and employee loans to ensure employees work with peace of mind, reduce turnover and strike rates, and produce higher organizational commitment among employees. Hsu (2010) proposed that companies use employee assistance to implement effective strategies in anticipation that it can improve employee morale and organizational commitment and enable employees to identify with the company’s goals and values and increase their loyalty to the company. Furthermore, because EAPs are mainly concerned with human resource development, their implementation can not only reduce turnover but also enable the company to improve its organizational performance and mitigate negative impacts from organizational change. Studies have also shown that the importance of and satisfaction with EAP implementation are significantly positively correlated with organizational commitment (Hsu, 2010). Hence, the introduction of an EAP by companies is a project for the long-term sustainable development of companies and the career development of their employees, which can influence employee perceptions of an corporate identity, and EAPs are worthy of corporate promotion (Lin, 2009; Chang, 2011). Employees are crucial assets of an enterprise; hence, enterprises should create favorable working environments that conform to the content of EAPs as well as employee expectations and perceptions of the program, which in turn affects employee work commitment, satisfaction, and performance (Yang et al., 2012).

In summary, EAPs serve as valuable tools for organizations to foster employee well-being, increase organizational commitment, and achieve long-term sustainable development. Their strategic implementation and promotion are crucial for creating supportive work environments and enhancing employee engagement and performance.

#### 1.1.2 Employee

Lin and Hu (2012) held that workplace stress tests are basic tools for enterprises to perform stress prevention management, and by providing relevant consulting services they can alleviate stress problems among employees and thus assist in establishing a service model for enterprise stress management. Therefore, through EAP implementation, enterprises can assist employees with physiological, psychological, family, and work problems, and further reduce their work stress. Hung (2008) discovered that the assistance programs provided by an organization influence employee work stress, job burnout, and interpersonal relationships in the workplace only when the EAPs improved the emotional intelligence of employees. Lin (2009) determined that companies with a high level of EAP implementation can effectively mitigate employee complaints about work and interpersonal relationships as well as health, household, financial, and other problems, with interpersonal problems having the largest share. Therefore, EAPs can provide diversified welfare measures to solve or alleviate the family, life, and work problems of employees and enable them to reconcile their body, mind, and spirit and to achieve family harmony, life satisfaction, and smooth work. Using EAP implementation to help employees exhibit stable emotions and behaviors can thereby enable them in achieving stable work performance and a high sense of commitment (Hsu, 2010). Employee well-being has been demonstrated to have a significant positive correlation with task performance and contextual performance; in other words, implementing EAPs improves employee well-being, thereby improving their work performance (Lin, 2008). Lin (2015) observed a positive correlation between EAP and employee well-being. EAPs have an inspirational element in their correlation with well-being, in that the stronger the service provided by an EAP is, the higher the personal happiness of employees is. Huang (2017) contended that timely interventions through EAPs effectively relieve employee emotional labor and further reduce their turnover intention. Therefore, EAPs have a moderating effect on the impact of emotional labor on turnover intention.

EAPs are instrumental in promoting employee well-being, managing stress, improving work performance, and reducing turnover intention within organizations. Their diverse services and interventions contribute to creating a supportive work environment and fostering employee satisfaction and commitment.

#### 1.1.3 Public sector

Kuo (2007) perceived an EAP to expect a hybrid in-house and outsourced model developed under the principles of independence, professionalism, dedicated responsibility, and confidentiality that only served employees during the trial period and provided diversified assistance with regard to aspects of work, life, and health. Sung (2009) was responsible for the promotion of an EAP during his tenure in the former Department of Health (presently the Ministry of Health and Welfare), Executive Yuan—the national authority on health matters. He combined the resources of the Department of Health and discovered that his colleagues generally anticipated strengthening their own correct understanding of “psychological counseling” as well as reinforcing the concept of “prevention is better than cure”. Shi (2009) adopted the study of a public sector EAP as an example to explore the effectiveness and operational results of current public sector EAPs and proposed the following four suggestions as a reference for future EAP implementation: an EAP is a welfare measure provided by the government for public employees; the revision of the “Plan for Promoting EAPs for School Employees of Central Authorities Under the Executive Yuan” should be managed by the Civil Service Housing and Welfare Committee or by competent authorities formulating inter-entity supply contracts; strengthen EAP promotion and establish the concept of correct use among colleagues to prevent the good intentions of the government regarding public service employees from being compromised; and discover the actual needs of public service employees, and develop EAPs meeting them. In addition, Chuan (2012) commented that the factors limiting implementation success in various public sector organizations include planning inflexibility and failure to adapt to local conditions; being limited to the planning framework set out by the Executive Yuan; insufficient resources; and lack of clear evaluation indices. The primary task for agencies promoting EAPs was to depart from the existing framework and develop customized EAPs for separate agencies based on organizational characteristics. Chiang (2012) examined the EAP policies of the Taichung Municipal Government and obtained the following five conclusions: EAP policies should be human resource management policies rather than only employee welfare policies; the internal–external integrated model is the optimal operating mechanism; comprehensive and convenient EAP policy establishment and provision of clear service procedures are recommended; service content should be adjusted and existing resources integrated; and tracking and feedback evaluation mechanisms should be established. Hsu et al. (2015) determined that employee EAP perceptions, service item satisfaction, and service effectiveness have all attained a level of satisfaction, with service effectiveness having the highest level. At the same time, the influence of EAP perception on service item satisfaction, that of service item satisfaction on service effectiveness, and that of EAP perception on service effectiveness were all significantly positive. Lin et al. (2017) argued that people’s perception of EAP service satisfaction and organizational commitment has reached a level of satisfaction, with service satisfaction with psychological counseling being the highest. In addition, it significantly influences organizational commitment, with the perceived influence of value commitment being the highest.

Overall, these studies highlight the importance of tailored EAP models, clear policies, and comprehensive service offerings in promoting employee well-being and organizational commitment. They also underscore the need for ongoing evaluation and adaptation to ensure the effectiveness of EAPs.

#### 1.1.4 Other

Hsiao (2003) suggested that most companies implement EAPs through the human resource department, use an internal volunteer system, welfare committees, and labor departments, and coordinate external resources or employ integration methods in implementation. In a case study, researchers determined that organizations attach substantial importance to supervisor interview skills, counselor communication guidance, preparation of personnel manuals, and new employee training, which produce considerable results (Chen et al., 2004). Wilson et al. (2004) proposed that when a company implements practical policies on work–life balance, they can significantly improve the organization’s safety climate, employee health, and employee perception of organizational support. Another study that examined the impact of formal organizational family support such as flexible work planning, bereavement care, child care, eldercare, psychological counseling and referral, family health insurance services, and financial assistance on employee well-being revealed that formal organizational family support effectively reduced employee stress and willingness to leave and simultaneously improved employee life satisfaction (Thompson and Prottas, 2006). Regarding EAP functions, human resources personnel can create a healthy work environment, increase employee value, and assist employees in connecting with existing resources; in terms of impact on the organization, EAPs can enhance corporate image and employee identification with the organization, promote favorable interaction between employees and their supervisors, and improve the quality of work (Lee, 2005). Employee use of EAP counseling markedly increases when the EAP is promoted (Lin and Wang, 2009). Finally, Yang (2008) uncovered that EAP satisfaction significantly influences work engagement, work–life balance, and enhancement of personal life.

A review of the aforementioned research confirms that EAPs can effectively address various problems, such as the work stress, number of employee complaints, absenteeism, and turnover rate (in the work aspect); common workplace interpersonal relationship problems (daily life aspect), and anxiety, unstable emotions and behavior, and medical expenses (health aspect). Furthermore, implementing EAPs can be inferred to markedly improve employee psychological well-being, work performance, organizational identification, daily life, and health, as well as have sustainable, comprehensive, favorable effects on organizations.

Overall, the research suggests that EAPs effectively address various problems including work stress, interpersonal issues, and health concerns. Implementing EAPs can significantly improve employee psychological well-being, work performance, organizational identification, daily life, and health, resulting in sustainable and favorable effects on organizations.

## 2 Methodology

### 2.1 Research procedure

This study first categorizes the service measures of employee assistance programs outlined by the Ministry of Labor in the “Employee Assistance Program Implementation Manual” into three major dimensions: work-related, life-related, and health-related, comprising a total of 22 service measures. These serve as the evaluation criteria for employee assistance programs in the public sector. Subsequently, a modified Delphi method is employed for expert questionnaire analysis, followed by the use of fuzzy analytic hierarchy process to explore the weights of evaluation criteria for employee assistance programs across different organizational types within the public sector. Finally, the research results are presented, research conclusions are summarized, and specific practical recommendations are provided.

### 2.2 Research participants

To understand the appropriateness of the EAP measures implemented in the public sector, this study used the four types of public sector organizations specified by the Directorate-General as the research scope, namely, the Executive Yuan, municipal governments, county and city governments, and others (consisting mainly of assemblies). Specifically, implementors of EAPs in the human resource departments of these types of organizations and the service target group of regular employees were adopted as the research subjects. Questionnaires were administered to at least 20 persons in each group, in the hope of understanding the current status of EAP implementation in the public sector from multiple perspectives. To enhance the value of the research results for future reference, we invited experts who had received awards based on the evaluation results of plans and those who have actively promoted an EAP but have not yet received awards to participate in order to increase the scope.

In all, 22 valid questionnaires were collected from the Executive Yuan group, 22 valid questionnaires from the municipal government group, 20 valid questionnaires from the county and city government group, and 17 valid questionnaires from the “others” group, for a total of 81 valid questionnaires.

### 2.3 Research instruments

**Figure 1:**
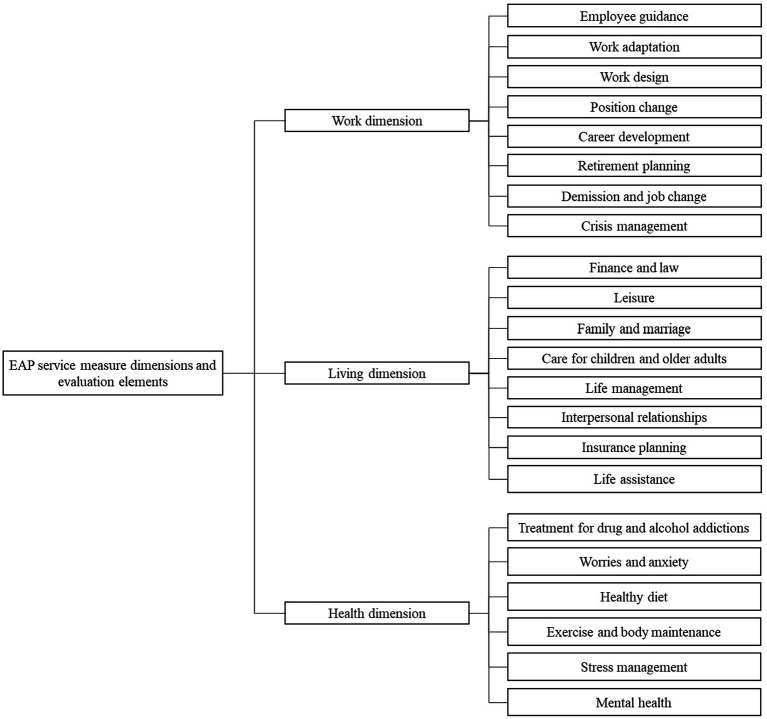
Initial draft of the evaluation criteria index framework for EAPs implemented by public sectors.

Assessing the implementation of EAP measures by the Ministry of Labor, a modified Delphi questionnaire combining the three aspects of EAPs, namely, work, life, and health, was used to establish a hierarchical relationship of the indices of EAP service measures and draft the index elements of the evaluation criteria for EAP service measures implemented by the public sector. The hierarchy of the indices comprised three layers— the final goal, EAP service measure dimensions, and evaluation elements—as shown in Figure 1.

#### 2.3.1 Modified Delphi questionnaire

An EAP implemented by the Ministry of Labor was analyzed to determine the connotation of EAP measures implemented by the public sector, and an initial draft of index elements for public sector EAP measures was compiled. A questionnaire was then formulated using a modified Delphi technique wherein experts were invited to evaluate the priority of each item. In general, Likert scales were used, and after the questionnaires were collected, statistical methods such as mode, median, and quartile were used to analyze the level of agreement and priority among the experts regarding the various classification items. A continuous integration process was then adopted, until the expert opinions converged and the index elements for EAP measures implemented by the public sector was finally derived.

In this study, a Likert 7-point scale was used in the modified Delphi questionnaire. Experts were invited to complete the questionnaires in paper or electronically and, according to the method proposed by Faherty (1979), the items were included in the next stage, namely, the fuzzy analytic hierarchy process (FAHP) questionnaire stage, when the interquartile range was less than 0.50, indicating an item had reached a high level of consistency.

#### 2.3.2 Fuzzy analytic hierarchy process questionnaire

According to the established hierarchical structure diagram, pairwise comparison items were designed. Experts were then asked to rate the importance and priority of each element. After asking the experts and scholars to rate the relative importance of the indices in each level, the weighting of each evaluation item was analyzed. A 9-point equidistant scale was used in the questionnaire for scoring, with *equally important*, *somewhat important*, *moderately important*, *extremely important*, and *absolutely essential* being assigned a value of 1, 3, 5, 7, and 9, respectively; values of 2, 4, 6, and 8 were used between the five basic anchors as intermediate measures. The questionnaire was filled by comparing the relative importance of the left and right elements.

### 2.4 Data processing and analysis

We used the 22 service measures of an EAP implemented by the Ministry of Labor as the basis for index construction. Subsequently, an FAHP questionnaire was administered to collect and summarize the original data to facilitate subsequent data analysis.

1. First, the collected questionnaires were input into Microsoft Excel, and the responses of each questionnaire were checked. After the data were verified, each valid questionnaire was coded and the values were inputted. Next, SuperDecisions software was used to perform FAHP statistical analysis.
2. The consistency ratio (CR) presented by each collected questionnaire was checked. This data represented whether a respondent’s answer was consistent and met the required standard. CR ≤ 0.1 indicated consistency and that the results could be used to establish a comparison matrix and calculate the relative weight of the subsequent element index.
3. The questionnaire items that passed the consistency test and were determined valid were converted from a pairwise comparison of positive reciprocal matrices into fuzzy positive reciprocal matrices. Using Microsoft Excel, the geometric means were calculated and integrated, and a pairwise matrix table of all responses was created.
4. The SuperDecisions software was used to analyze the pairwise comparison matrix table according to each index level to obtain the relative weight of each group of indices. Because the weight value of each index was a fuzzy value, the data were exported to Microsoft Excel to perform defuzzification and normalization of the triangular fuzzy weight values. The indices were then ranked in terms of importance and converted into statistical charts to present and explain the results in order to construct the weights of the evaluation criteria.

## 3 Results and discussion

### 3.1 Expert background data analysis

A total of 22 experts participated in the Executive Yuan group. Most (73%) were women, most had a college or junior college degree (59%), and most had worked at their current agency for less than 3 years (55%). A total of 23% of the experts were currently persons-in-charge of EAPs, managing 1.4 projects on average. Those with experience using EAP measures accounted for 23% of the experts, with the work aspect accounting for 80% of the measures, followed by the daily life aspect (20%).

The municipal government group contained a total of 22 experts. Among them, 77% were women, 77% had a college or junior college degree, and 68% had worked in their current agency for less than 3 years. A total of 32% of the experts were currently persons-in-charge of EAPs; they executed 1.14 projects on average. Experts who had experience using EAP measures accounted for 23% of the total, with the work aspect accounting for 56% of the measures, followed by the daily life aspect (33%) and the health aspect (11%).

In the county and city government group, a total of 20 experts participated. Among them, most were women (85%), most had a college or junior college degree (80%), and half had worked at their current agency for 4–10 years (50%). A total of 55% of the experts were currently persons-in-charge of EAPs, directing 3.45 projects on average. In all, 35% of the experts had experience using EAP measures, with the work aspect accounting for 40% of the measures, followed by the daily life aspect (33%) and the health aspect (27%).

A total of 17 experts participated in the “others” group. Most (53%) were women, 82% had a college or junior college degree, and 47% had worked in their current agency for 4–10 years. A total of 29% of the experts were currently persons-in-charge of EAPs, managing 1.2 projects on average. Experts who had experience in using EAP measures accounted for 24% of the total, with the work aspect accounting for 50% of the measures, followed by the daily life and health aspects (25% each).

### 3.2 Modified Delphi questionnaire

#### 3.2.1 Executive Yuan group

In the work aspect, employee needs in the Executive Yuan group concerned six items, namely, employee guidance, job adaptation, job transition, career development, retirement planning, and crisis management; in the daily life aspect, their needs concerned six items, specifically, financial and legal advice, leisure and entertainment, family and marriage, childcare and eldercare, interpersonal relationships, and living assistance; as for the health aspect, they were five areas pertaining to anxiety, healthy diet, exercise and health maintenance, stress management, and mental health. Summarizing this information indicated that employees in the Executive Yuan group required 17 service measures, and five items, namely, job design, resignation and career change, life management, insurance planning, and drug and alcohol abstinence, could be deleted.

#### 3.2.2 Municipal government group

Regarding the work aspect, employee needs in the municipal government group concerned seven items, namely, employee guidance, job adaptation, job design, job transition, career development, retirement planning, and resignation and career change; in the daily life aspect, their needs concerned six items, specifically, financial and legal advice, leisure and entertainment, family and marriage, childcare and eldercare, interpersonal relationships, and living assistance; as for the health aspect, they were five matters pertaining to drug and alcohol abstinence, anxiety, healthy diet, exercise and health maintenance, and mental health. To summarize this information, employees in the municipal government group required 18 service measures, and four items, specifically, crisis management, insurance planning, living assistance, and stress management, could be deleted.

#### 3.2.3 City and county government group

For the work aspect, employee needs in the city and county government group concerned eight items, namely, employee guidance, job adaptation, job design, job transition, career development, retirement planning, resignation and career change, and crisis management; in the daily life aspect, their needs concerned five items, specifically, financial and legal advice, childcare and eldercare, life management, interpersonal relationships, and living assistance; as for the health aspect, these were five matters pertaining to drug and alcohol abstinence, anxiety, healthy diet, exercise and health maintenance, and stress management. Summarizing this information revealed that employees in the city and county government group required 18 service measures, and four items—leisure and entertainment, family and marriage, insurance planning, and mental health—could be deleted.

#### 3.2.4 Others group (consisting mainly of assemblies)

In the work aspect, employee needs in the others group concerned eight items, namely, employee guidance, job adaptation, job design, job transition, career development, retirement planning, resignation and career change, and crisis management; in the daily life aspect, their needs concerned six items, specifically, financial and legal advice, leisure and entertainment, family and marriage, interpersonal relationships, insurance planning, and living assistance; as for the health aspect, they were five matters pertaining to anxiety, healthy diet, exercise and health maintenance, stress management, and mental health. Summarizing this information shows that employees in the city and county government group required 19 service measures, and three items, namely, childcare and eldercare, life management, and drug and alcohol abstinence, could be deleted.

### 3.3 Fuzzy analytic hierarchy process questionnaire

#### 3.3.1 Index of the EAP measures implemented in the executive Yuan group

The EAP measures of the Executive Yuan group focused mainly on the health aspect (relative weight = 0.41921), followed by the daily life (0.31599) and work aspects (0.26479). The CR values of the work, daily life, and health aspects were 0.02, 0.01, and 0.06, respectively, and were all less than 0.10, indicating a consistent overall evaluation process. The departments and employees of the Executive Yuan group emphasized EAP measures that concerned health; particularly, anxiety was deemed the most crucial (relative weight = 0.27646), which was markedly higher than the other four measures of the health aspect, namely, exercise and health maintenance (0.24082), healthy diet (0.18712), mental health (0.17433), and drug and alcohol abstinence (0.12126). In the daily aspect, interpersonal relationships (0.21378) was the most vital, followed by family and marriage (0.17361), financial and legal advice (0.16849), childcare and eldercare (0.16844), living assistance (0.14828), and leisure and entertainment (0.12739). As for work, employee guidance (0.19209) was emphasized, followed by career development (0.18893), job adaptation (0.182), job transition (0.17253), retirement planning (0.14411), and crisis management (0.12034).

#### 3.3.2 Index of the EAP measures implemented in the municipal government group

The EAP measures of the municipal government group focused mainly on the work aspect (relative weight = 0.53225), followed by health (0.24704) and daily life (0.22071). The CR values of the work, daily life, and health aspects were 0.02, 0.03, and 0.03, respectively, all less than 0.10, indicating a consistent overall evaluation process. Among the EAP measures for the aspect of work, employee guidance (0.26767) was emphasized most, followed by job adaptation (0.21988), job transition (0.13862), job design (0.10699), career development (0.09831), retirement planning (0.08947), and resignation and career change (0.07906). Regarding health, anxiety (0.26707) was the most essential, followed by stress management (0.25443), mental health (0.17679), exercise and health maintenance (0.15362), and healthy diet (0.1481). As for the daily life aspect, measures were prioritized in the descending order of financial and legal advice (0.24682), family and marriage (0.20917), childcare and eldercare (0.20406), leisure and entertainment (0.12688), life management (0.10707), and interpersonal relationships (0.10599).

#### 3.3.3 Index of the EAP measures implemented in the county and city government group

The EAP measures of the county and city government group focused mainly on work (relative weight = 0.51138), followed by daily life (0.28158) and health (0.20704). The CR values of the work, daily life, and health aspects were 0.02, 0.02, and 0.01, respectively, and thus all were less than 0.10, indicating a consistent overall evaluation process. Among the EAP measures for the work aspect, employee guidance (0.22887) was most emphasized, followed crisis management (0.13748), job transition (0.12574), job adaptation (0.12315), retirement planning (0.1197), career development (0.10901), job design (0.10208), and resignation and career change (0.05397). Regarding the daily life aspect, childcare and eldercare (0.38034) was the most vital, followed by interpersonal relationship (0.22327), financial and legal advice (0.1571), life management (0.12899), and living assistance (0.1103). As for the health aspect, stress management (0.33183) was the most central, although the difference in the relative weights of the remaining measures, namely, anxiety (0.25721), exercise and health maintenance (0.15898), healthy diet (0.14265), and drug and alcohol abstinence (0.10933), was small.

#### 3.3.4 Index of the EAP measures implemented in the “others” group

The EAP measures of the “others” group were similar to those of the municipal government and county and city government groups, in that they mainly focused on the work aspect. However, the relative weight for the “others” group was 0.41049, which was substantially less than that of the municipal government and county and city government groups. The relative weight of the health aspect was 0.38956, which was markedly higher than the two aforementioned groups, whereas that of the daily life aspect was 0.2. The CR values of the work, daily life, and health aspects were 0.01, 0.02, and 0.02, respectively, all less than 0.10, indicating a consistent overall evaluation process.

In the “others” group, the difference in the relative weights of the various measures for the work aspect was small. In descending order, the relative weights were crisis management (0.21537), retirement planning (0.16621), career development (0.14634), employee guidance (0.1319), job adaptation (0.1204), job design (0.10414), resignation and career change (0.06612), and job transition (0.04953). Regarding health, drug and alcohol abstinence (0.412) was the most prominent, followed by mental health (0.24713), stress management (0.21304), exercise and health maintenance (0.19155), healthy diet (0.17528), and anxiety (0.17299). As for the daily life aspect, financial and legal advice (0.22713) was the most crucial. The difference in the relative weights of the remaining measures, namely, interpersonal relationship (0.18226), living assistance (0.18075), family and marriage (0.16752), insurance planning (0.13505), and leisure and entertainment (0.10729), was small.

Based on the aforementioned results, we can infer that employees have specific needs in the evaluation criteria of the EAPs and service measures implemented and that these needs differed among organizations. Even when the evaluation criteria of two organizations had the same priority, elements of the service measures differed. Therefore, the results indicated that each group has its own specific needs regarding EAP service measures, and the evaluation criteria must be adjusted depending on the type of organization.

## 4 Conclusion and suggestions

### 4.1 Conclusion

According to the analysis results of the modified Delphi expert questionnaire in this study, it was found that across the four organizational structures within the public sector, there is a variation in the required employee assistance program measures. Overall, the highest demand was observed for job-related assistance measures, followed by those related to personal life, with health-related measures being the least in quantity. However, the specific content and quantity of required employee assistance program measures varied among the different organizational structures. Due to the differing employee needs in the four types of organizational structures within the public sector, the content of the implemented employee assistance program measures also differs, leading to variations in the constructed evaluation criteria for employee assistance programs in the public sector.

#### 4.1.1 Work aspect

##### 4.1.1.1 Employee guidance

The Executive Yuan, municipal government, and county and city government groups all emphasized employee guidance the most. For new employees, the mentorship guidance mechanism of appointing a colleague with 2 years of experience and is familiar with the department’s business to guide new employees is an extremely crucial service measure, in which understanding of the organizational culture and job adaptation can be accelerated through guidance on practical operations and administrative workflows. This result is similar to that of Chen et al. (2004). Implementing the mentorship mechanism, conducting training courses or self-growth workshops for new employees, and providing them with diverse written or online electronic guidance information shorten the time for new employees to familiarize themselves with the new work. This is a measure that should not be underestimated.

##### 4.1.1.2 Crisis management

The “others” group viewed crisis management as the most essential, which indicated that local legislative assemblies more frequently interacted with legislators compared with other organizations. The work problems faced by the employees are less routine and more urgent and thus require quick responses. Hence, establishing a set of standard operating procedures for managing major crises or incidents and adopting fast communication, information transmission, and notification methods to facilitate the handling of emergency responses is an EAP service measure that employees particularly need. This result is the same as that of Hung (2014). Preparing workflow planning, memos, and multiple drills in advance can strengthen the ability of employees to respond quickly and appropriately to crises, and support in managing them is indeed the most essential item in the work aspect of the “others” group.

#### 4.1.2 Daily life aspect

##### 4.1.2.1 Interpersonal relationships

In the Executive Yuan group, interpersonal relationships were prioritized, indicating favorable interpersonal relationships being a need of employees. Organizations can arrange courses on workplace interpersonal relationships and communication to guide employees in establishing favorable interpersonal relationships with colleagues, supervisors, the public, and legislators to prevent workplace bullying and emotional blackmail. In addition, workshops related to interpersonal issues can be organized to assist employees in developing desirable interaction and communication methods and in solving interpersonal relationship problems in their daily lives. This result is similar to that in a case study conducted by Csiernik et al. (2001), where interpersonal relationships were ranked second among consultation events, and other scholars have expressed similar views (Selvik et al., 2004; Hung, 2008). Departments in the Executive Yuan group are responsible mainly for the planning of national policies. Favorable interpersonal relationships affect policy communication and the effectiveness of policy promotion. Therefore, using EAPs to enhance employee skills in establishing favorable interpersonal relationships is the primary need for the daily life aspect.

##### 4.1.2.2 Financial and legal advice

The municipal government and the “others” groups emphasized financial and legal advice. Financial pressure is a major source of stress that affects the mental health, productivity, absenteeism, and alcohol addiction of employees (Jacobson et al., 1996; Peirce et al., 1996). This suggests that assisting employees in handling financial planning such as tax filing, tax reduction, retirement planning, and debt consolidation prevents the work performance of employees from being affected by economic instability and other factors. Such assistance can be provided through lectures on financial discipline or through financial consulting services such as personal financial planning and debt management and negotiation. Common legal problems among employees include problems related to traffic accidents, the sale of fixed and personal assets, marriage, and inheritance. By matching lawyers who can satisfy the legal needs of employees through the internal legal department of the organization or the bar association, contracts and opportunities for cooperation can be negotiated to provide related services. This is similar to the opinion of Lin (2009). Financial planning and legal advice are needs that demand high professionalism. Hence, the municipal government and others should provide financial and legal advice channels to assist employees with financial and legal problems, the most crucial element in the daily life aspect of employees.

##### 4.1.2.3 Childcare and eldercare

The county and city government group prioritized childcare and eldercare. Employees require their organizations to provide temporary care spaces for their children or older family members. Childcare institutions could improve services to include after-school transportation, hiring qualified childcare staff, and after-school learning services, talent training courses, and summer camps. If no idle space is available for setting up a nursery or kindergarten, organizations can contract specific nurseries to provide discounts on childcare services, tuition and fees, book purchases, and infant products as well as form agreements with long-term care facilities for older adults. Doing so could benefit the family members of employees in multi-generational households by making appropriate care services available, hence preventing related matters from affecting employee work efficiency. This is consistent with the findings of Wilson et al. (2004) and Thompson and Prottas (2006). Providing the family members of employees with childcare and eldercare services to ensure employees can work with less worry is certainly the most crucial EAP service item. Hence, county and city government organizations should emphasize the planning of childcare and eldercare in their EAPs concerning the daily life aspect.

#### 4.1.3 Health aspect

##### 4.1.3.1 Anxiety

The Executive Yuan and municipal government groups ranked anxiety as the most vital element, which suggests that many employees are faced with the problem of anxiety. Hence, employees could use testing services for mental health (such as anxiety and emotional management) provided by the EAP of their organization. After a test is completed, scores can be calculated and the results explained to the employee. When necessary, the test results can be explained to employees one-on-one with therapists to address anxiety problems. This is similar to the findings of Chang (2011). Prevention should be emphasized over cure; hence, the Executive Yuan and municipal government groups should be made aware of this and prioritize measures for basic prevention such as increasing employee psychological capital.

##### 4.1.3.2 Stress management

The county and city government group prioritized stress management, suggesting that the organizations can use stress test questionnaires to understand the stress level of colleagues and supervisors. Based on the questionnaire results, measures of the aspect required by the employees can be collected and coordinated with short, medium, and long-term stress management programs, such as stress management workshops, relaxation breathing training, stress inoculation training, yoga, and aerobic exercise. Such measures can help employees face varying levels of work and daily life stress to take appropriate stress management actions based on their personal stress levels and to engage in effective stress management activities. Providing stress tests as an EAP service measure and then offering relevant effective stress management activities according to the employee needs ameliorates employee stress problems. This is similar to the findings of Mark et al. (2003) and Lin and Hu (2012). Although appropriate levels of stress enable people to realize their potential, excessive stress leads to counterproductive results. The health aspects of the county and city government group should be focused on service measures for employee pressure adjustment and management. Services measures in the health aspect of the EAPs provided by the county and city government group should focus on stress management.

##### 4.1.3.3 Mental health

In the “others” group, mental health was prioritized. Organizations can provide individual professional psychological counseling services by establishing internal counseling units or collaborating with outside professional psychological counseling institutions to assist public employees in dealing with personal problems. Awareness seminars can be regularly conducted or pamphlets distributed to inform employees about relevant information and to build a sense of trust. Through monthly, quarterly, or annual reports, organizations can also discern the types of problems and service satisfaction of their employees, and the reports can serve as references for future service adjustments or organizational management. The present results indicate EAPs should establish internal psychology counseling units or collaborate with outsourced psychological consulting institutions to provide individual consulting to assist employees in managing personal problems. Hence, lectures, individual consultations, workshops, and group counseling can be adopted to teach employees to improve self-awareness and establish correct mental health concepts. Sung (2009) derived similar empirical findings. Mental health education is the secondary prevention method for mental health. Organizations in the “others” group should screen employees for psychological problems for primary prevention and testing and provide psychological counseling channels to address the physiological and psychological stress problems of employees early on.

In summary, the results indicate that discerning how best to implement EAPs based on the type of public sector organization to improve the physical and mental health of employees to enable them to work with a healthy body and mind and thus improve personal and organizational performance has become a crucial topic that cannot be ignored by government organizations. However, the evaluation criteria regarding the execution of EAPs have not been sufficiently customized according to organization type and EAP model, rendering it impossible to clearly match the needs of the respective organization employees. Hence, the effects of EAP implementation are yet to be clearly presented.

### 4.2 Suggestions

On the basis of the results of this study, we propose the following suggestions and hope that the various viewpoints expressed can provide references for the Directorate-General of Personnel Administration of the Executive Yuan when promoting EAPs in the public sector and thus enhance the effectiveness of the EAPs.

#### 4.2.1 Executive Yuan group

This group is the competent authority in the central government under the Executive Yuan and has relatively more resources in terms of financial budget and labor than other government organizations. The results of this study indicate that employees emphasize anxiety in the health aspect of their concerns. We suggest establishing outsourced EAP service models through which government organizations collaborate with external professional EAP organizations to provide employees with diversified services, protect employee privacy, and reduce employee mistrust when using them. External service models are suitable for organizations in the Executive Yuan group with clear employee needs, supportive supervisors, and a sufficient budget.

#### 4.2.2 Municipal government group

Municipal governments play an intermediary role in governing. Although their resources may not be as abundant as those of the Executive Yuan group, they are more sufficient than those of county and city governments. This study determined that municipal government employees prioritized employee guidance in the work aspect. Hence, we recommend that an internal–external integration model be adopted as the optimal operation model. Commissioned professional organizations and internal departments in charge can be responsible for the planning and promotion of the core work of the EAPs. This is most suitable for municipal governments that have mature internal resource integration, high participation among departments, and experience in promoting programs and outsourcing EAPs.

#### 4.2.3 County and city government group

County and city governments are relatively small in scale (among government bodies) and have fewer resources. The results of the present study suggest that employees in this group most emphasized employee guidance in the work aspect. Therefore, we recommend using a single internal–external integration model or a dual integration model with a resource network model as the operation model. Establishing EAP policies in a comprehensive manner meeting employee needs, providing clear service procedures, adjusting the integration of service content and existing social resources, and adopting the “integration mode and resource connection mode” to adjust their use is best. Accordingly, establishing comprehensive EAP policies that meet employee needs with clear service procedures and regulating the integration of service content with existing resources through adopting a dual integration and resource network model could be an excellent approach.

#### 4.2.4 “Others” group

Compared with the other three organization types, with the exception of the central bank, the remaining organizations were legislative assemblies that are small in scale and lack resources. Hence, we recommend that these organizations adopt “coordination between resource networks” models or single-service models as the operation model. Coordinating resource network models with single-service models and taking into account employee needs and EAP quality when faced with realistic, objective, environmental conditions is also an innovative and flexible response approach.

These conclusions indicate that the evaluation criteria of the four types of public sector organizations should be revised based on factors such as employee needs and the type of EAP service model adopted. For example, the evaluation criteria for the EAPs of the Executive Yuan group prioritized anxiety (of the health aspect), and outsourced EAP service models could be adopted. The municipal government group focused on the employee guidance element of the work aspect, and an integration model could be adopted. The county and city government group also highlighted employee guidance, but here a dual integration and resource network model could be adopted. In the “others” group, crisis management (of the work aspect) was prioritized, and a coordinated resource network and single-service model could be used.

### 4.3 Practical implication

In light of the diverse nature of public sector organizations, it is imperative to tailor evaluation methodologies and criteria to suit their individual characteristics. The Directorate-General of Personnel Administration of the Executive Yuan should initiate a comprehensive review of the existing evaluation framework for EAPs, acknowledging the varying needs, workforce sizes, resource capacities, and service delivery models across different entities.

One proposed approach involves conducting detailed assessments to identify the unique challenges and requirements of each organization. This may entail collaborating closely with department heads, human resource specialists, and frontline employees to gather insights into the specific stressors, work-life balance issues, and mental health concerns prevalent within their respective domains.

Subsequently, a flexible and adaptable evaluation system should be devised, allowing for customized performance metrics that align with the organizational objectives and priorities. This could involve establishing key performance indicators (KPIs) tailored to measure the effectiveness of EAP interventions in addressing identified needs, enhancing employee well-being, and optimizing operational outcomes.

Moreover, streamlining administrative procedures and paperwork associated with EAP evaluations is paramount to alleviate the burden on program administrators and facilitate smoother implementation processes. By simplifying documentation requirements and minimizing bureaucratic hurdles, resources can be redirected toward more impactful initiatives aimed at enhancing service quality and organizational performance.

Furthermore, fostering a culture of continuous improvement and learning within public sector entities is essential to maximize the long-term benefits of EAPs. Regular feedback mechanisms, performance reviews, and stakeholder consultations should be integrated into the evaluation framework to ensure ongoing refinement and adaptation in response to evolving organizational dynamics and employee needs.

Ultimately, by adopting a tailored approach to evaluating EAPs that accounts for the diverse needs and contexts of public sector organizations, the Directorate-General can foster a more conducive environment for employee well-being, organizational effectiveness, and national competitiveness. This strategic realignment of evaluation practices holds the potential to yield significant dividends in terms of service quality enhancement and overall performance improvement across the public sector landscape.

### 4.4 Future research recommendations

This study focused on personnel in the human resources departments responsible for implementing employee assistance programs in various organizational structures within the public sector, including central administrative agencies of the Executive Yuan, municipalities and counties, as well as other units (primarily councils). Although the breadth of the research scope was considered, limitations such as human resources and time constraints restricted the inclusion of all public sector employees as research subjects. Therefore, the inferences drawn from this study are limited. In the future, expanding the scope of research to include a broader range of public sector employees and utilizing big data analysis techniques could enable rapid and precise analysis of service measures and indicators for employee assistance programs across different organizational structures within the public sector. This would facilitate the development of more objective evaluation criteria, maximizing the academic and practical value of such research.

The evaluation criteria for employee assistance programs in the public sector constructed in this study were derived from the 22 service measures proposed by the Ministry of Labor, confirmed through the Delphi method and fuzzy analytic hierarchy process. However, it is possible that other influencing factors are not fully accounted for in these evaluation criteria. Therefore, it is recommended that future research consider revising the evaluation criteria for employee assistance programs based on the specific attributes of the public sector.

## Data availability statement

The datasets presented in this article are not readily available because the participants did not give their consent for making the data publicly available. Requests to access the datasets should be directed to corresponding author.

## Ethics statement

Ethical review and approval was not required for the study on human participants in accordance with the local legislation and institutional requirements. Written informed consent was not required to participate in this study in accordance with the local legislation and institutional requirements.

## Author contributions

Y-CC: Formal analysis, Methodology, Writing – original draft, Writing – review & editing. S-CC: Resources, Writing – original draft. H-CC: Conceptualization, Validation, Writing – review & editing.
